# New Perspectives on Escherichia coli Signal Peptidase I Substrate Specificity: Investigating Why the TasA Cleavage Site Is Incompatible with LepB Cleavage

**DOI:** 10.1128/spectrum.05005-22

**Published:** 2023-04-26

**Authors:** Joanna E. Musik, Jessica Poole, Christopher J. Day, Thomas Haselhorst, Freda E.-C. Jen, Thomas Ve, Veronika Masic, Michael P. Jennings, Yaramah M. Zalucki

**Affiliations:** a Institute for Glycomics, Griffith University, Gold Coast, Queensland, Australia; University of Guelph College of Biological Science

**Keywords:** LepB, secretion, signal peptidase I, substrate specificity, TasA

## Abstract

Escherichia coli signal peptidase I (LepB) has been shown to inefficiently cleave secreted proteins with aromatic amino acids at the second position after the signal peptidase cleavage site (P2′). The Bacillus subtilis exported protein TasA contains a phenylalanine at P2′, which in B. subtilis is cleaved by a dedicated archaeal-organism-like signal peptidase, SipW. We have previously shown that when the TasA signal peptide is fused to maltose binding protein (MBP) up to the P2′ position, the TasA-MBP fusion protein is cleaved very inefficiently by LepB. However, the precise reason why the TasA signal peptide hinders cleavage by LepB is not known. In this study, a set of 11 peptides were designed to mimic the inefficiently cleaved secreted proteins, wild-type TasA and TasA-MBP fusions, to determine whether the peptides interact with and inhibit the function of LepB. The binding affinity and inhibitory potential of the peptides against LepB were assessed by surface plasmon resonance (SPR) and a LepB enzyme activity assay. Molecular modeling of the interaction between TasA signal peptide and LepB indicated that the tryptophan residue at P2 (two amino acids before the cleavage site) inhibited the active site serine-90 residue on LepB from accessing the cleavage site. Replacing the P2 tryptophan with alanine (W26A) allowed for more efficient processing of the signal peptide when the TasA-MBP fusion was expressed in E. coli. The importance of this residue to inhibit signal peptide cleavage and the potential to design LepB inhibitors based on the TasA signal peptide are discussed.

**IMPORTANCE** Signal peptidase I is an important drug target, and understanding its substrate is critically important to develop new bacterium-specific drugs. To that end, we have a unique signal peptide that we have shown is refractory to processing by LepB, the essential signal peptidase I in E. coli, but previously has been shown to be processed by a more human-like signal peptidase found in some bacteria. In this study, we demonstrate how the signal peptide can bind but is unable to be processed by LepB, using a variety of methods. This can inform the field on how to better design drugs that can target LepB and understand the differences between bacterial and human-like signal peptidases.

## INTRODUCTION

An essential process in bacteria is the secretion of proteins across membranes. There are many secretion pathways in bacteria; however, the most common is the general secretion pathway (Sec). A typical secretion pathway is as follows: (i) targeting the protein to the membrane, (ii) translocation of the protein across the membrane, and (iii) releasing the protein from the membrane (1). Secreted proteins contain an N-terminal signal peptide which assists in directing the protein through the Sec secretion pathway. Once the protein has been secreted across the membrane, the signal peptide is removed by a signal peptidase (SPase), allowing the protein to be either incorporated into the membrane or released into the periplasmic space.

The signal peptide can be defined by three conserved regions: (i) the positively charged N terminus, (ii) the hydrophobic core, and (iii) the C-terminal hydrophilic region containing the signal peptide cleavage site (1). However, in addition to the signal peptide, the early mature region of the protein also plays a role in the efficiency of secretion, such as a general net-negative charge (2–6), an absence of proline at P1′ (7, 8), and aromatic amino acids at P2′ (9–11).

The signal peptide of nonlipoproteins is removed by signal peptidase I and is an essential step in the process. Signal peptidase I can be separated into two main types, P type and ER type, generally found in prokaryotes and eukaryotes, respectively (12). Although their functions are very similar, the structure and mechanism of these two types of SPase I are quite different, with the active site dyad being the biggest difference between the two (13): P-type SPase I contains a Ser-Lys dyad (12), while the ER type contains a Ser-His dyad (14).

Previously, we examined a set of 143 verified LepB-cleaved Escherichia coli proteins and reported that none had an aromatic amino acid present two amino acids after the cleavage site (P2′) (15). Studies with maltose binding protein (MBP) in which aromatic amino acids were substituted at P2′ showed this to lead to an increase in unprocessed precursor protein by the P-type signal peptidase, LepB, in E. coli (9). In addition, we have also shown that the Bacillus subtilis protein TasA, which contains a phenylalanine at P2′ and is processed by a dedicated, ER-type signal peptidase, SipW (16), is toxic when expressed in E. coli (10). This toxicity is due to blocking the Sec pathway because the TasA signal peptide cannot be processed by the E. coli SPase I, LepB (10). This study demonstrated that the residues surrounding the predicted TasA cleavage site (P3-P4′) are crucial to LepB inhibition, but the precise reason why these residues inhibit processing by LepB is unknown. Predictions by the SignalP algorithm (17) suggest there is nothing unusual about the signal peptide and that it should be cleaved by LepB (10). In this study, we have designed peptides based on the findings of Musik et al. (10) that mimic the TasA signal sequence, to act as probes to help understand the interaction leading to inefficient cleavage of TasA by LepB and thereby provide new information useful for the design of SPase I inhibitors.

## RESULTS

### Peptides of TasA-MBP-P2′ and MBP bind to LepB with similar affinities.

Our previous study demonstrated that when the predicted TasA signal peptide was fused to mature MBP or β-lactamase (both common model systems used to study bacterial secretion) at amino acid position P2′, the expressed fusion proteins accumulated unprocessed precursor material in E. coli (10). Most strikingly, overexpression of the TasA-MBP-P2′ and TasA-MBP-P1′ fusions led to cell death in E. coli (10). These studies narrowed down the amino acids responsible for the secretion-inhibiting activity to those between P3 and P4′ (TWA-AFND).

**FIG 1:**
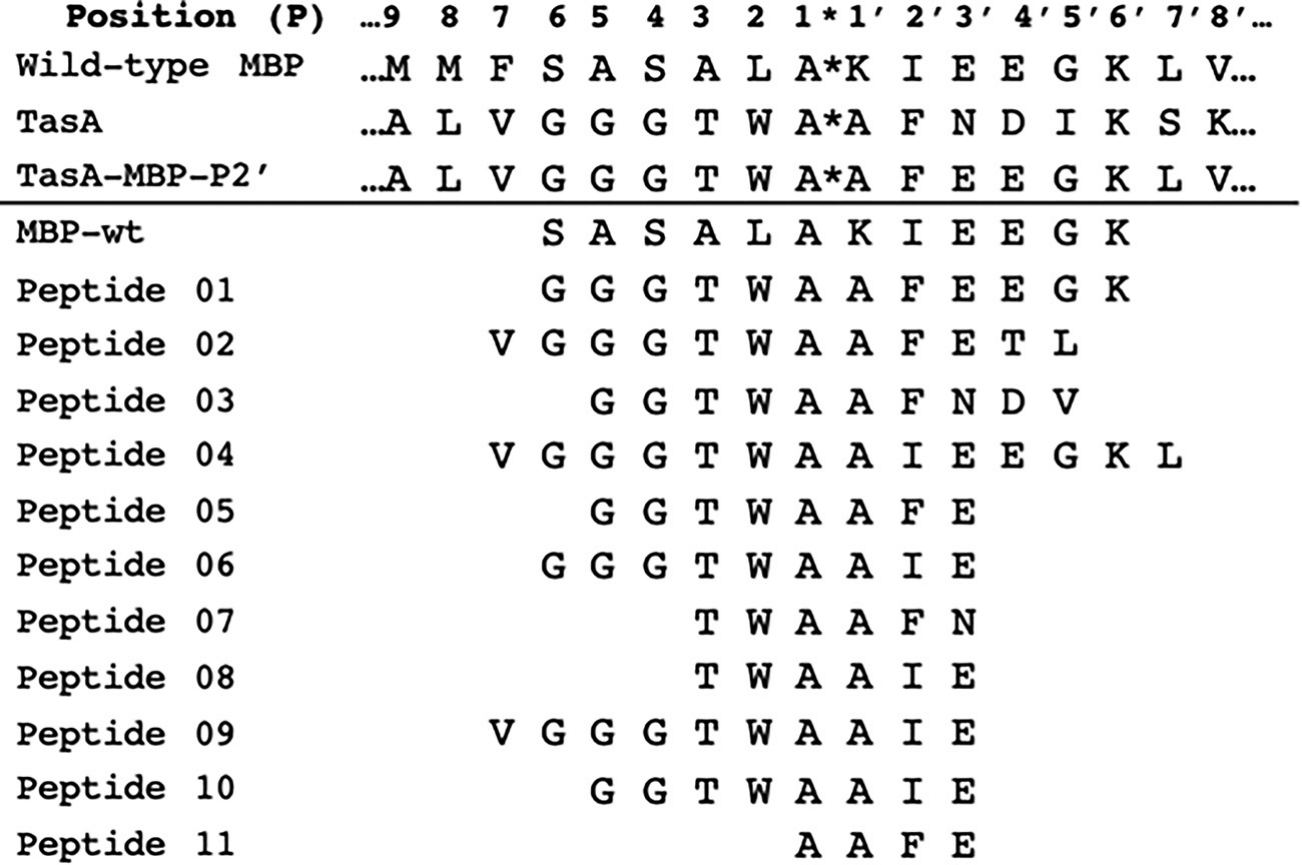
Synthetic peptides (below line) used in this study aligned with the wild-type MBP, TasA, and TasA-MBP-P2′ protein sequence (above line) predicted cleavage sites. *, predicted cleavage site.

Previously, we have shown by surface plasmon resonance (SPR) that LepB can bind to and release short peptides based on the signal peptide cleavage site (9). Using this SPR assay, we measured the direct interaction of a peptide based on the TasA-MBP-P2′ cleavage site (Fig. 1) with LepB and compared it to a similar-length peptide based on the MBP wild-type (wt) sequence. These peptides encompassed six amino acids either side of the SignalP-predicted cleavage site of both MBP (SASALAKIEEGK) and TasA-MBP-P2′ (GGGTWAAFEEGK, peptide 01). These peptides were caused to flow over LepB immobilized on an SPR biosensor chip, and their on- and off-rates and affinity were determined. The MBP wt peptide and peptide 01 had similar on-rates (2.41 × 10^3^ M^−1^ s^−1^ versus 4.44 × 10^3^ M^−1^ s^−1^) and off-rates (1.74 × 10^−2^ s^−1^ versus 4.34 × 10^−2^ s^−1^), resulting in similar affinities, 8.01 μM and 10.31 μM, respectively (Table 1; see Fig. S1L in the supplemental material).

**TABLE 1:** SPR on- and off-rates and IC_50_ data of peptides^*a*^

Peptide	Sequence	*K_a_* (M^−1^ s^−1^) on-rate	*K_d_* (s^−1^) off-rate	IC_50_ (mg/mL)
MBP-wt	SASALAKIEEGK	2.41 × 10^3^ ± 0.45 × 10^3^	1.74 × 10^−2^ ± 0.03 × 10^−2^	1.665
01	GGGTWAAFEEGK	4.44 × 10^3^ ± 0.41 × 10^3^	4.34 × 10^−2^ ± 1.51 × 10^−2^	1.929
02	VGGGTWAAFETL	1.14 × 10^3^ ± 0.12 × 10^3^	1.47 × 10^−2^ ± 0.35 × 10^−2^	1.218
03	GGTWAAFNDV	1.46 × 10^5^ ± 0.27 × 10^5^	9.47 × 10^−1^ ± 1.10 × 10^−1^	1.206
04	VGGGTWAAIEEGKL	2.64 × 10^4^ ± 0.88 × 10^4^	1.70 × 10^−1^ ± 0.21 × 10^−1^	2.338
05	GGTWAAFE	ND	ND	0.810
06	GGGTWAAIE	1.20 × 10^3^ ± 0.04 × 10^3^	2.08 × 10^−2^ ± 0.07 × 10^−2^	3.891
07	TWAAFN	6.04 × 10^4^ ± 1.19 × 10^4^	3.21 × 10^−1^ ± 0.52 × 10^−1^	1.394
08	TWAAIE	2.50 × 10^3^ ± 0.23 × 10^3^	2.39 × 10^−3^ ± 1.25 × 10^−3^	0.446
09	VGGGTWAAIE	3.34 × 10^3^ ± 0.68 × 10^3^	5.91 × 10^−3^ ± 0.28 × 10^−3^	0.761
10	GGTWAAIE	4.77 × 10^3^ ± 0.59 × 10^3^	4.21 × 10^−3^ ± 0.49 × 10^−3^	0.389
11	AAFE	1.59 × 10^4^ ± 0.20 × 10^4^	4.42 × 10^−1^ ± 1.20 × 10^−1^	1.656

a ND, not determined, due to peptide solubility issues. The mean absorption rate (*K_a_*) and dissociation rate (*K_d_*) ± standard error are from triplicate data.

### Peptides based on TasA and the TasA-MBP fusion bind to LepB with differing binding kinetics.

To identify what specific region of the TasA, TasA-MBP, and TasA-Bla cleavage site could be involved in inhibiting processing by LepB, we ordered 31 peptides of low purity and of various lengths. These peptides were screened to identify the ones which bound best to LepB via SPR and inhibited LepB activity via a fluorescence resonance energy transfer (FRET) assay (data not shown). From this analysis, the 10 best peptides were ordered at high purity to further probe which amino acids are important for the TasA-mediated LepB inefficient cleavage phenotype (Fig. 1). Of the 10 peptides, six bound at a similar on-rate (range of 1.14 × 10^3^ to 4.77 × 10^3^ M^−1^ s^−1^) (Table 1) as the MBP wt peptide (2.41 × 10^3^ M^−1^ s^−1^) (Table 1) and four were at least 10-fold faster (1.59 × 10^4^ M^−1^ s^−1^ to 1.46 × 10^5^ M^−1^ s^−1^) (Table 1). Our hypothesis that LepB activity is inhibited by the TasA-MBP and TasA-Bla fusions via slow release by LepB would be supported by the TasA signal sequence mimic peptides having a lower off-rate than the MBP wt peptide in the SPR assay. Three of the peptides, 08, 09, and 10, had an off-rate at least 10-fold lower than the MBP wt peptide (1.74 × 10^−2^ s^−1^) (Table 1). The peptide that was released the slowest was peptide 08 (TWAAIE; 2.39 × 10^−3^ s^−1^), followed by peptide 10 (GGTWAAIE; 4.21 × 10^−3^ s^−1^) and peptide 09 (VGGGTWAAIE; 5.91 × 10^−3^ s^−1^), which were all based on the TasA-MBP-P1′ fusion (Table 1). This indicates that replacing the phenylalanine at P2′ with isoleucine leads to slower release by LepB. Hence, the phenotype we observed, whereby expression of the TasA-MBP-P1′ peptide led rapidly to cell death (10), may be explained by the slower release by LepB. These results encouraged us to continue to test and develop inhibitory peptides based on the TasA signal sequence.

### TasA peptides competitively inhibit LepB activity.

While the SPR assay above was able to show the relative kinetics of binding of the TasA signal sequence peptide mimics to LepB, we wanted to determine whether these peptides could directly inhibit LepB activity. To test if the peptides were able to competitively inhibit the cleavage of a typical signal sequence peptide by LepB, and to determine the efficiency of this inhibition, we utilized a quantitative FRET-based enzyme assay (18). The cleavage of the fluorescent peptide, Dabcyl-DAPPAKAA-Edans, by LepB leads to an increase in relative fluorescence over time. If the ability of LepB to cleave this peptide is inhibited, the increase in relative fluorescence per second should decrease in a concentration-dependent manner. LepB Δ2-76 was purified from inclusion bodies for this experiment as the His-SUMO tag on commercially available LepB used in our SPR studies was nonconducive to FRET assays. We were able to purify active LepB Δ2-76 at a high purity using a protocol modified from previous publications (18, 19) (see Materials and Methods and Fig. S4).

**FIG 2:**
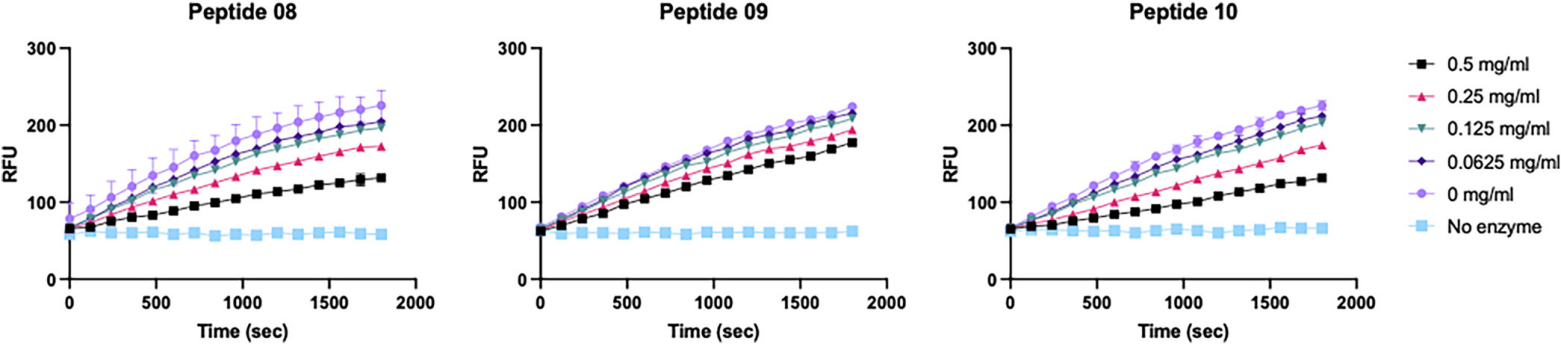
LepB kinetic enzyme assay in the presence of indicated concentrations of either peptide 08, 09, or 10. Data points are averages from 3 biological replicates; error bars are ±1 standard deviation (SD). RFU, relative fluorescent units.

We observed inhibition of LepB activity with a subset of the peptides (Table 1 and Fig. S2). The best inhibitors were peptides 08 (TWAAIE), 09 (VGGGTWAAIE), and 10 (GGTWAAIE), which had 50% inhibitory concentrations (IC_50_s) of 0.446, 0.761, and 0.389 mg/mL, respectively (Fig. 2 and Table 1). This correlates well with the SPR data, as these three peptides all had both a higher on-rate and lower off-rate than the MBP wt peptide substrate (Table 1). These peptides contain the motif TWAAIE, suggesting that this region (P3 to P3′) is important to inhibit LepB cleavage.

### Tryptophan at P2 in TasA-MBP-P2′ peptide blocks the active site serine of LepB.

To see if we could identify a structural reason why the TasA signal peptide inhibits processing by LepB, we undertook molecular modeling of TasA-MBP-P2′ (GGGTWAAFEE) peptide against the X-ray crystal structure of LepB (PDB 1B12) (20) using the flexible peptide docking algorithm CABS-dock web server (21).

**FIG 3:**
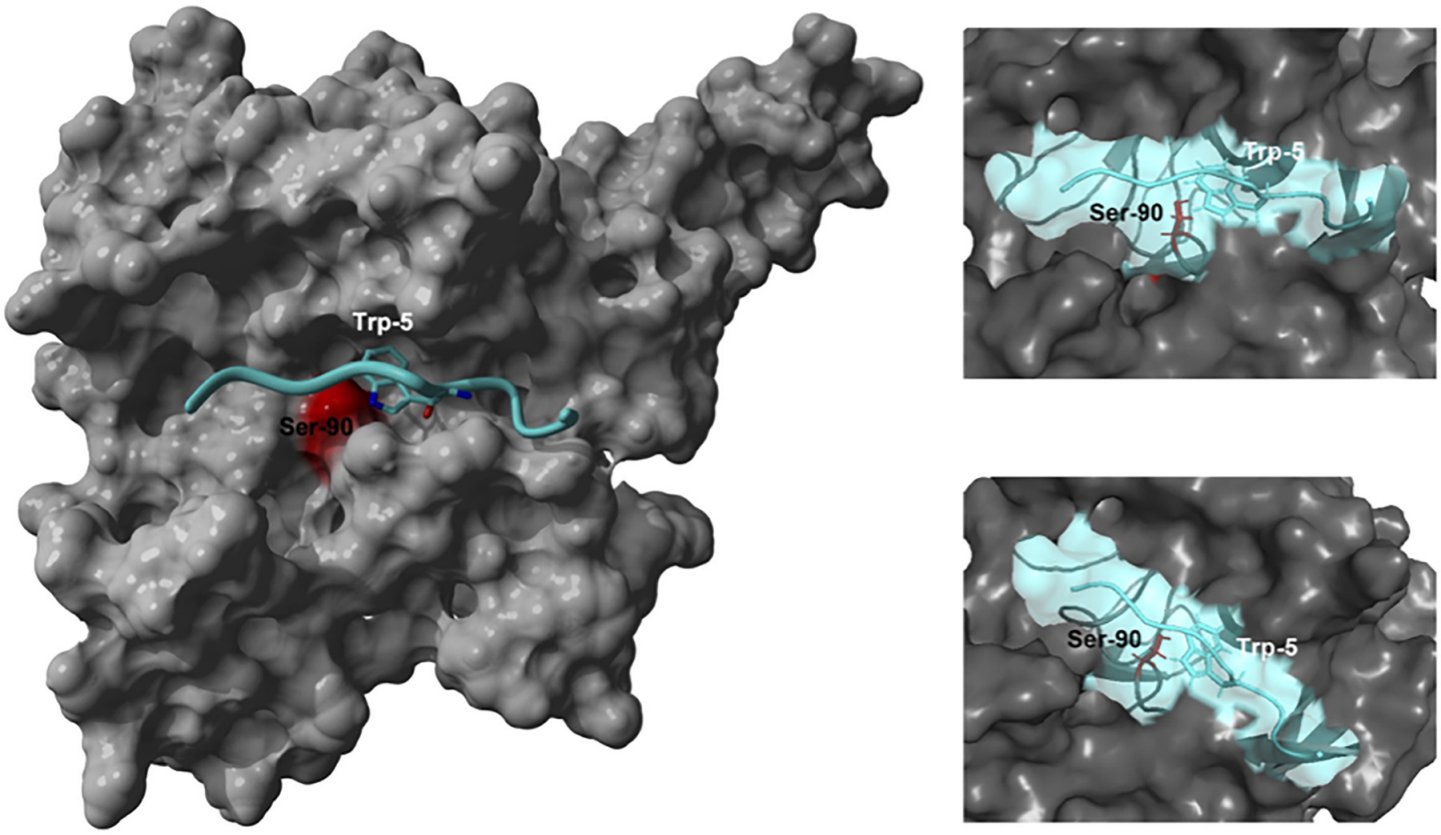
Molecular modeling of the interaction between TasA-MBP peptide (GGGTWAAFEE) and LepB Δ2-76. The peptide is displayed in a ribbon structure, with the Trp-5 side chains highlighted. The active site residue, Ser-90, of LepB is highlighted in red. The images depict the entire enzyme-TasA-MBP complex (on the left), a close-up view of the cleavage site (on the top right), and LepB Δ2-76 rotated 45 degrees clockwise (on the bottom right).

One thousand structures were generated and clustered using the root mean square deviation (RMSD) values in the top 10 models. The LepB structure with a modeled surface representation demonstrates a narrow channel with Ser-90 in the center (Fig. 3). Interestingly, the best energy model of P2 docked into TasA shows that the tryptophan (Trp-5) at the P2 position of the peptide anchors itself into this narrow channel with strong interactions with Ser-90, the active site amino acid of LepB (Fig. 3).

### Changing the P2 tryptophan residue to alanine in TasA-MBP-P1′ alleviates growth inhibition and leads to significantly more mature protein.

**FIG 4:**
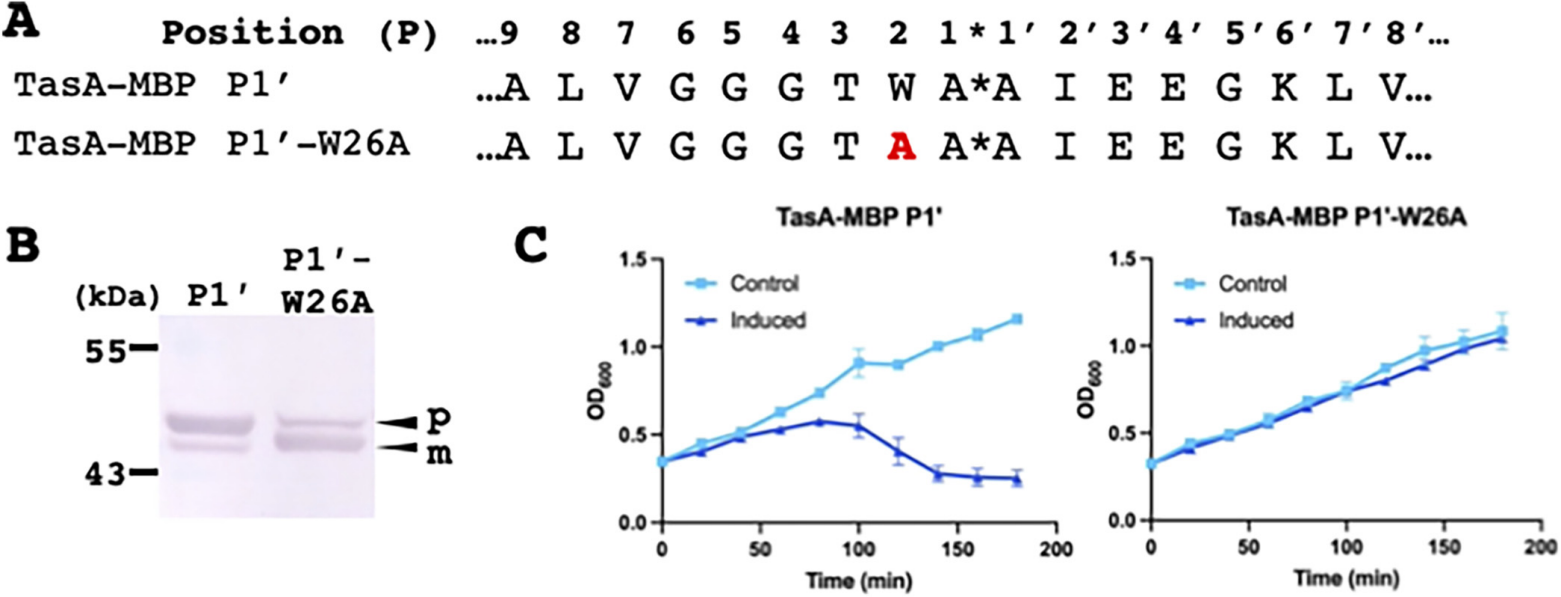
(A) TasA-MBP-P1′ and TasA-MBP-P1′-W26A sequence. *, predicted SPase I cleavage site. (B) TasA-MBP-P1′ and TasA-MBP-P1′-W26A anti-MBP whole-cell lysate after a 30-min induction on a Western blot. P, precursor protein; m, mature protein. (C) Growth curve of TasA-MBP-P1′ and TasA-MBP-P1′-W26A, induced with IPTG at *t* = 0. Data are averages from 3 biological replicates; error bars are ±1 standard deviation.

The molecular modeling indicates that the tryptophan at P2 could be responsible for the slower processing of the TasA signal peptide by LepB. To test this experimentally, we changed the P2 tryptophan in the TasA-MBP-P1′ fusion protein (Fig. 4A) to alanine. The TasA-MBP-P1′ fusion protein was chosen for these studies as this fusion protein had the most severe growth defect and the most unprocessed signal peptide in our previous study (10) and the lowest off-rate as measured by SPR (Table 1). When TasA-MBP-P1′-W26A (Fig. 4A) was overexpressed in media, the cell death phenotype was no longer present (Fig. 4C). We also observed evidence of improvement in the processing of this protein, with much less precursor present on a Western blot compared to TasA-MBP-P1′ (Fig. 4B and Fig. S3). Hence, we have experimental confirmation that the removal of the P2 tryptophan of TasA-MBP-P1′ can improve the processing by LepB (Fig. 4).

### TasA peptides have potential for development as new LepB targeting antibiotics.

We have demonstrated that peptides 08, 09, and 10 (Fig. 1) are released slowly from LepB and can inhibit LepB activity in an *in vitro* assay (Table 1). Expression of TasA can kill E. coli presumably via LepB inhibition (10), and it is well established that LepB activity is essential for bacterial viability (22, 23). We tested the two best TasA mimic peptides, 08 (TWAAIE) and 10 (GGTWAAIE), as well as the smallest peptide, 11 (AAFE), for growth inhibition/killing using an MIC assay. The MIC assay was first conducted against E. coli in both rich and minimal media, supplemented with or without a subinhibitory concentration of dimethyl sulfoxide (DMSO) to assist the peptides in permeating the membrane. No MIC was observed for all peptides against E. coli. The MIC assay was then conducted against Staphylococcus aureus strains FDA209 and USA300 in rich medium, where peptide 11 (AAFE) was able to inhibit growth in the presence of subinhibitory DMSO with an MIC of 1 mg/mL against both strains.

## DISCUSSION

Previously, we have presented the deadly phenotype of the B. subtilis protein TasA when expressed in E. coli, both as a full-length protein and with its signal peptide and early mature protein region fused onto MBP and Bla (10). The toxic effect of this protein was first thought to be due to the presence of the aromatic amino acid phenylalanine at P2′ of TasA, as, in general, the presence of aromatic amino acids at P2′ results in inefficient signal sequence processing in E. coli (9). However, we determined that other amino acids surrounding the cleavage site, i.e., from P3 to P4′, were also inhibiting TasA processing by LepB.

To further investigate the reason for the slower processing of TasA by LepB, we undertook two separate experiments using peptide mimics of the TasA signal sequence: SPR to probe peptide-LepB binding kinetics and a LepB enzyme assay to measure peptide inhibitory activity directly. From an earlier screen of peptides that inhibited LepB activity, we subsequently analyzed 11 peptides, one based on TasA-MBP-P2′ to assess initial binding kinetics and the additional 10 based on the TasA and TasA fusion protein sequences to further probe the TasA-LepB interaction (Fig. 1), and tested their ability to bind to LepB as well as competitively inhibit LepB. The SPR binding results demonstrated that they had broadly similar or higher on-rates; three of these peptides had an off-rate more than 10-fold lower than that of MBP wt peptide (peptides 08, 09, and 10) (Table 1). These peptides were based on the TasA-MBP-P1’ fusion protein (Fig. 4A), which also had the most extreme growth inhibition phenotype when expressed in E. coli in our previous study (10). These data are consistent with the toxic phenotype of expressed TasA and the TasA-MBP-P2′ and TasA-MBP-P1′ fusion proteins in E. coli being due to their tight interaction with LepB, outcompeting other Sec-dependent secreted proteins and titrating out the available LepB, leading to the cell death phenotype (10).

To further investigate the precise reason for the inefficient interaction of the TasA-MBP-P2′ peptide with LepB, we undertook peptide docking experiments showing that the tryptophan at P2 in the TasA-MBP-P2′ peptide anchors the peptide into a narrow channel of LepB and therefore blocks the active site serine-90 of LepB. Previously, we reported that a point mutation of the P2 tryptophan (Fig. 1), obtained in a directed evolution experiment using TasA-Bla-P2′ fusion protein, led to improvement in the processing of this fusion protein (10). The tryptophan was predominately replaced by smaller residues (alanine and glycine), leading to almost complete recovery in the processing of precursor to mature protein as seen on a Western blot. In the present study, replicating the same P2 tryptophan substitution using the TasA-MBP-P1′ fusion protein, we observed a pronounced loss of the toxic phenotype with no change in growth rate when the protein was overexpressed, as well as a reduction in the amount of accumulated precursor protein present on the Western blot (Fig. 4). These experimental results support the conclusions drawn from the molecular modeling of the TasA-LepB interaction, which indicated that a tryptophan at P2 may hinder the processing of the signal peptide due to its large size and proximity to the active site serine-90 residue on LepB. Second, the modeling also indicates that the phenylalanine at P2′ sits directly above the active site serine-90 (Fig. 3). We have previously shown that aromatic residues were absent at P2′ in 143 verified LepB-cleaved signal peptides (15) and that adding aromatic residues at P2′ to MBP led to the increase in unprocessed signal peptide (9). The modeling results suggest the proximity of P2′ phenylalanine to the active site serine-90 could lead to slower processing of the signal peptide. However, further experiments are needed to resolve exactly how the P2′ aromatic amino acid hinders LepB processing.

It is interesting that the tryptophan at P2 in the TasA signal peptide inhibits processing by LepB as there has been no reported bias against any amino acids at this position. The classic cleavage motif is Ala-X-Ala, stating that any amino acid should be accommodated at the second position before the cleavage site. Although no bias against tryptophan has been previously reported, Choo and Ranganathan (24) noted that histidine, tryptophan, and tyrosine are clearly underrepresented in all signal peptides from P10 to P10′. From our previous data set of 143 verified E. coli SPase I-processed signal peptides, eight contain a tryptophan at P2, so this residue alone is not detrimental for LepB processing of signal peptides. It is most likely the tryptophan, in conjunction with other residues in the TasA signal peptide, that leads to a conformation in which the tryptophan inhibits serine-90 activity of LepB, leading to slower processing. In this regard, the TasA signal peptide is an exciting tool, as it can clearly be processed by an ER-type SPase I, SipW (16). Learning precisely how this peptide inhibits LepB processing could lead to future design of inhibitors that specifically target bacterial SPase I and not eukaryotic signal peptidases.

We briefly assessed the ability of these peptides, unmodified, to inhibit growth of E. coli and S. aureus via an MIC assay. It has previously been shown that the SPase I in S. aureus, SpsB, is able to complement LepB activity in E. coli (25), and inhibitors that target LepB have also been shown to inhibit SpsB (26). We observed no inhibition of E. coli by these peptides, which could be due to many factors such as inability to cross the outer membrane or hydrolysis by peptidases in the bacteria, or they could have aggregated or broken down before reaching the target (27). However, peptide 11 (AAFE) was able to kill S. aureus at 1 mg/mL. It is likely that this peptide was able to reach S. aureus SPase I due to its small size. However, further experiments are required to determine the mechanism of how peptide 11 kills S. aureus and whether it is through inhibition of its signal peptidase, SpsB.

The common sequence shared by the three peptides (08, 09, and 10) that demonstrated the most inhibition of LepB via FRET is TWAAIE (Fig. 1), which is the entire sequence of peptide 08. This peptide had an approximately 10-fold-lower off-rate to LepB as measured by SPR. The TWAAIE motif encompasses the P3 to P3′ section of the TasA-MBP-P1′ fusion protein sequence, which is very close to our previous conclusion that the P3 to P4′ region is important for the inefficient cleavage of the protein (10). What makes these three peptides have better inhibitory potential than some of the other peptides is not clear, as two others also contain the TWAAIE sequence, such as peptide 04 (VGGGTWAAIEEGKL) and peptide 06 (GGGTWAAIE), and they did not have good inhibitory activity, with IC_50_s of 2.338 and 3.891 mg/mL, respectively. Peptide 06 (GGGTWAAIE) differs from both peptide 09 (VGGGTWAAIE) and peptide 10 (GGTWAAIE) by one residue at the N terminus. Hence, changes in this region (P4 to P6) could impact how strongly they bind to LepB, and this space should be explored when further developing these peptides.

Here, we have demonstrated the importance of the P2 tryptophan in TasA and TasA fusion proteins as being the reason for their inefficient cleavage by LepB. We have also shown that peptides based on these sequences are able to bind and be released at a lower rate by LepB, and they could competitively inhibit the binding of other LepB substrates. Further development of these peptides should focus on the N-terminal region and where it interacts with LepB, maintaining the TWAAIE motif, and enabling the peptides to better permeate the bacterial membranes. With further development and modifications of these peptides, they could become inhibitors of signal peptidase I activity.

## MATERIALS AND METHODS

### Molecular cloning techniques.

Cloning was carried out in E. coli DH5α [F^−^ (80d*lacZ*ΔM15) (*lacZYA-argF*)*U169 hsdR17* (r^−^ m^+^) *recA1 endA1 relA1 deoR*]. Protein expression was carried out in E. coli BL21(DE3) [*dcm ompT hsdS*(r_B_^−^ m_B_^−^) *gal*]. Ampicillin was used at 100 μg/mL. All PCRs were carried out using KOD (Novagen, Madison, WI, USA; catalog no. 71086). Ligations using T4 DNA ligase were performed according to manufacturer’s instructions (New England Biolabs [NEB], Ipswich, MA, USA). DNA sequencing was done using the BigDye Terminator method (Griffith University DNA Sequencing Facility, Nathan, QLD, Australia, and Australian Genome Research Facility, Melbourne, VIC, Australia).

### Construction of LepB Δ2-76.

LepB was amplified from E. coli strain DH5α using LepB_R (CAGATAGGATCCTCAGTGATGGTGATGGTGATGGATGCCGCCAATGCGACTTAAGCG) and LepB_F (GATATACCATGGTGCGTTCGTTTATTTATGAAC) primers using KOD polymerase, a proofreading enzyme. The forward primer was designed to remove the first 75 residues of LepB. The PCR product and the plasmid pET15b were both digested with NcoI and BamHI and then ligated and transformed into strain DH5α. Prospective clones were miniprepped and digested with BamHI and NcoI, and clones with an insert were sequenced to confirm that pET15b-LepBΔ2-76-His was correct. The His tag was then removed via inverse PCR using the primers LepB2-76_F (ATGGATGCCGCCAATGCGACTTAAGC) and LepB2-76_R (TGAGGATCCGGCTGCTAACAAAGCC). The PCR product was ligated and transformed into strain DH5α. Prospective clones were miniprepped and were sequenced to confirm that pET15b-LepBΔ2-76 was correct. This plasmid was then transformed into E. coli BL21(DE3) for expression.

### LepB Δ2-76 purification.

LepB Δ2-76 (28, 29) was purified using a modification of methods previously described (18, 19). E. coli BL21(DE3) expressing pET15b-LepB Δ2-76 was grown at 37°C in M9 minimal medium containing 0.1% Casamino Acids. Once the culture reached an optical density at 600 nm (OD_600_) of 0.8, protein expression was induced with 0.5 mM isopropyl-β-d-thiogalactopyranoside (IPTG), and the culture was grown for a further 4 h. The cells were pelleted and then resuspended in 25 mM Tris-HCl, 5 mM NaCl, pH 7.4, and incubated for 30 min at 37°C with a protease inhibitor tablet (Merck, Kenilworth, NJ, USA; 11836170001), DNase, and lysozyme. The cells were run through a high-pressure homogenizer (Avestin, Ottawa, ON, Canada). The cells were then pelleted again to isolate the inclusion bodies, which were then washed in 20 mM Tris-HCl, 10 mM EDTA, 0.5% Triton X-100 four times using an 18-gauge (18G) drawing-up needle and mixed for 5 min, before being pelleted again and washed a further 3 times. The washed inclusion body pellet was then resuspended in 6 M guanidine in 20 mM Tris-HCl and left at 4°C overnight. The next morning, the LepB was diluted 2:1 in 25 mM Tris-HCl, 5 mM NaCl, containing 0.5% Triton X-100 and dialyzed against the same buffer at 4°C, changing the buffer every 12 h for 3 days, with the last 3 dialysis buffers containing no Triton X-100. After dialysis the LepB was centrifuged at 15,000 × *g* for 30 min to pellet any misfolded protein. The LepB Δ2-76 was >95% pure at this point and was then concentrated using a 10-kDa-molecular-weight-cutoff (MWCO) spin column to >10 mg/mL.

### SPR.

Surface plasmon resonance (SPR) analysis on a Pioneer FE system (Sartorius, Göttingen, Germany) was used to determine the on- and off-rates of custom peptides (Mimotopes, Mulgrave, VIC, Australia) with purchased LepB (Sapphire Bioscience, Redfern, NSW, Australia) (9). Briefly, LepB Δ2-76 His-SUMO was immobilized onto a CDH chip (Sartorius, Göttingen, Germany) onto flow cells 1 and 3 by an amine coupling reaction using 0.2 M 1-ethyl-3-[(3-dimethylamino)propyl]-carbodiimide (EDC) and 0.05 M *N*-hydroxysuccinimide (NHS). The EDC-NHS mix was caused to flow over at 15 μL/min for 100 s, LepB was injected at 10 μL/min for 10 min, and then ethanolamine was caused to flow over at 10 μL/min for 10 min. Flow cell 2 was used as a reference cell, and no LepB was injected over this cell. The assay was performed using a OneStep assay method with 75% of the loop. Peptides were caused to flow over the flow cells at a concentration of 50 μM at a flow rate of 50 μL/min with a dissociation period of 180 s. A regeneration cycle at a flow rate of 50 μL/min for 3 min with Tris-EDTA (TE) was completed after every cycle. Data were collected in triplicate, and analysis was completed with the Qdat analysis software (Sartorius, Göttingen, Germany; see Fig. S1 in the supplemental material).

### FRET.

The ability of the peptides to competitively inhibit another peptide was analyzed using fluorescence resonance energy transfer (FRET) (18). In this experiment we used the LepB Δ2-76 material we had purified because the His-SUMO tag of the purchased material affected the binding of the peptides. A reaction in a 100-μL reaction mixture in phosphate-buffered saline (PBS) (pH 7.4) containing 0.1% Triton X-100 was initiated by adding 450 nM LepB Δ2-76 to a black, clear-bottom 96-well plate (Corning, Corning, NY, USA). In triplicate, wells contained a 2-fold serial dilution of the peptide being tested from 0.5 mg/mL and 10 μM fluorescent peptide, Dabcyl-DAPPAKAA-Edans (Mimotopes, Mulgrave, VIC, Australia). While the peptide containing the two fluorophores is intact, the Edans emission is quenched by Dabcyl through FRET. However, once the peptide is cleaved by LepB, the fluorophores are no longer in close enough proximity for the transfer of energy and the fluorescence emission of Edans is recorded. The cleavage of the fluorescent peptide by LepB was recorded using an excitation wavelength of 340 nm and an emission wavelength of 490 nm recorded every 2 min for 30 min in a Tecan Infinite 200 Pro plate reader. Data were analyzed using the formula [(RFU_2_ − RFU_1_)/(time_2_ − time_1_)] = RFU/s on the linear portion of the reaction (where RFU stands for relative fluorescent units), and the absolute IC_50_ was determined using the simple linear regression analysis on Prism 9. Since no commercial LepB inhibitor currently exists, the control for 100% inhibition was achieved by not adding any LepB Δ2-76 to a well containing only the fluorescent peptide.

### Molecular modeling.

The modeling was performed using the CABS-dock web server for flexible protein-peptide docking (30). CABS-dock enables full flexibility of the peptide structure and large-scale flexibility of protein fragments during the blind search for a binding site. Several benchmark tests have been reported previously (21). The X-ray crystal structure (PDB 1B12) (20) was used as the target protein, and the TasA-MBP-P2′ amino acid sequence GGGTWAAFEE was used as ligand input. The standard CABS-dock web server procedure generates 1,000 model structures of the protein-peptide complex. Here, we used clustering based on the RMSD of the entire protein-peptide complex. The resulting structures are grouped in clusters of similar complexes and ranked according to cluster size from the largest to the 10th largest. Ten top-ranked CABS-dock models were visualized.

### Construction of TasA-MBP-P1′-W26A.

The tryptophan (W26) was changed to an alanine using inverse PCR on the TasA-MBP-P1′ template (10) using the primers W26A_F (GAAGAAGGTAAACTGGTAATCTGG) and W26A_R (AAATGCTGCGGCTGTTCCTCCTCCAACTAAAG). The inverse PCR product was phosphorylated using T4 polynucleotide kinase (PNK) (NEB), self-ligated with T4 ligase (NEB), and then transformed into strain DH5α. The W26A mutation was confirmed by sequencing using primer malE-upseq (CGGTTCTGGCAAATATTCTG).

### TasA-MBP-P1′ and TasA-MBP-P1′-W26A growth curve.

The TasA-MBP-P1′ and TasA-MBP-P1′-W26A proteins (Fig. 4A) were grown in E. coli DH5α overnight in LB broth supplemented with 10 μg/mL ampicillin and 0.2% l-glucose. Samples were measured at *A*_600_, and diluted to an optical density (OD) of 1. From these dilutions, 150 μL of cells was added to 3 mL of fresh growth medium, to give a starting OD reading of approximately 0.05. The cells were grown at 37°C with shaking, and measurements were taken every hour until mid-log phase. Then, the cultures were split (2 × 1.5 mL), and one was induced with 0.1 mM IPTG. Measurements were taken every 20 min for 3 h postinduction. All cultures were grown in triplicate.

### Expression of TasA-MBP-P1′-W26A.

Expression of TasA-MBP-P1′ or TasA-MBP-P1′-W26A (Fig. 4A) was done in E. coli DH5α. Briefly, cells from an overnight culture, grown in LB broth supplemented with 10 μg/mL ampicillin and 0.2% l-glucose, were subcultured 1:50 into fresh medium. Expression was induced by adding 0.1 mM IPTG at mid-log phase. After 30 min of induction, a 1-mL sample was spun down and resuspended in 1× Western loading buffer. The samples were normalized by OD_600_, and samples were run on a 4 to 12% Bis-Tris Bolt gel at 150 V for 2 h. The proteins were transferred to nitrocellulose and incubated with the primary antibody, anti-MBP (ThermoFisher, Waltham, MA, USA; PA1-989) after blocking in 5% skim milk in Tris-buffered saline with Tween 20 (TBS-T) and then incubated with secondary antibody, anti-rabbit IgG (Sigma, St. Louis, MO, USA; A3687), both at a 1:10,000 dilution. The Western blots were developed using a 5-bromo-4-chloro-3-indolylphosphate (BCIP)–nitroblue tetrazolium (NBT) solution.

### MIC assay.

The resistance to the peptides was determined using MIC methods as previously described (31). Briefly, 5 × 10^5^ cells were added to a 96-well plate which contained a peptide 2-fold serially diluted in either M9 minimal medium containing 5% DMSO and 0.2% glucose or Mueller-Hinton medium containing 5% DMSO. Plates were incubated overnight at 37°C, and the MIC result was scored as the last set of wells that contained no growth of bacteria. Bacteria tested were E. coli MG1655 containing an empty pBAD plasmid, E. coli BL21(DE3) containing an empty pET15b plasmid, S. aureus FDA209, and S. aureus USA300. Peptides 08 and 10 were tested from 0.5 mg/mL, and peptide 11 was tested from 1 mg/mL.
