# Supplementary material for: New Perspectives on Escherichia coli Signal Peptidase I Substrate Specificity: Investigating Why the TasA Cleavage Site Is Incompatible with LepB Cleavage
Source: Microbiol Spectr. 2023 Apr 26;11(3):e05005-22. doi: 10.1128/spectrum.05005-22 (PMC10269814; doi:10.1128/spectrum.05005-22)
Supplement: Supplemental file 1 — Fig. S1 to S4. Download spectrum.05005-22-s0001.pdf, PDF file, 0.7 MB [file spectrum.05005-22-s0001.pdf]

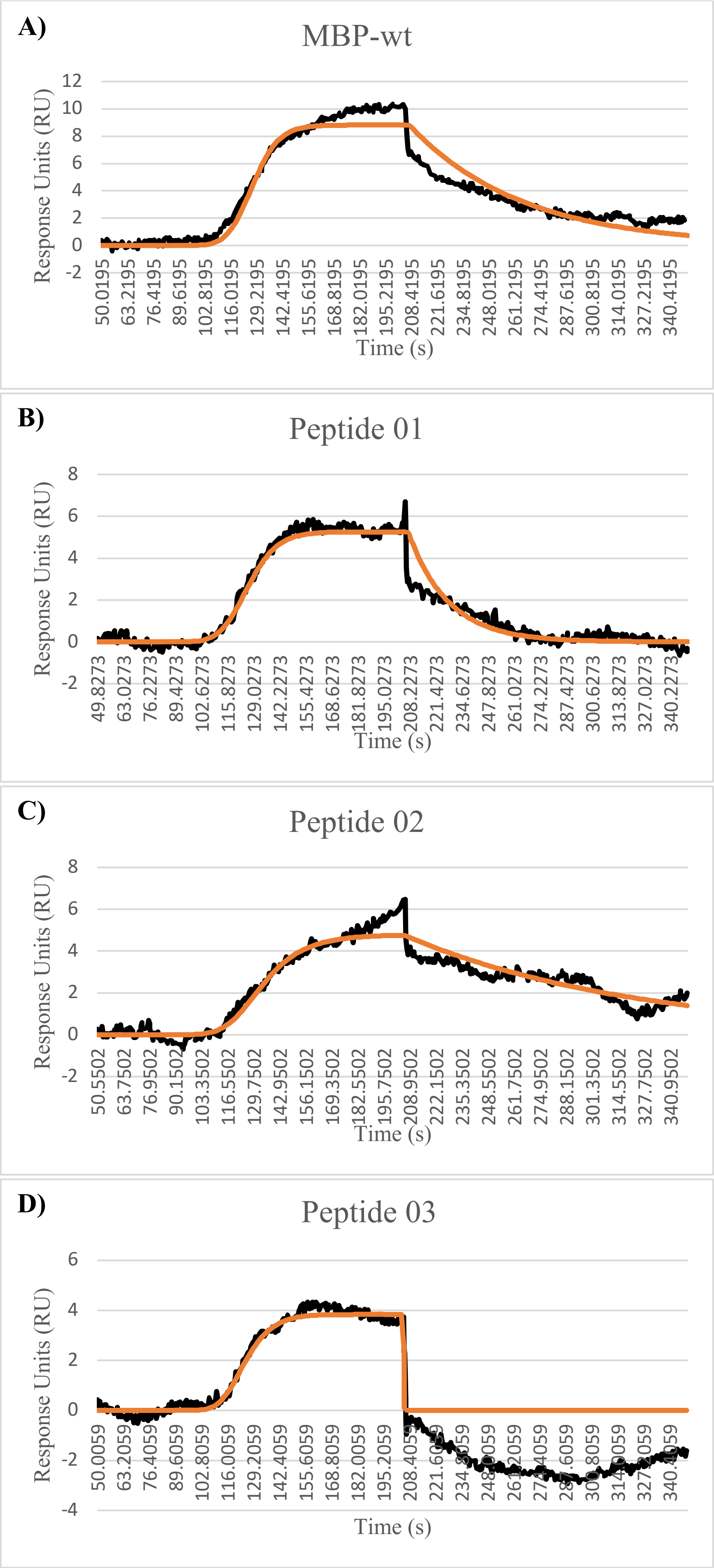
A)
MBP-wt
Response Units (RU)
Time (s)
B)
Peptide 01
Response Units (RU)
Time (s)
C)
Peptide 02
Response Units (RU)
Time (s)
D)
Peptide 03
Response Units (RU)
Time (s)

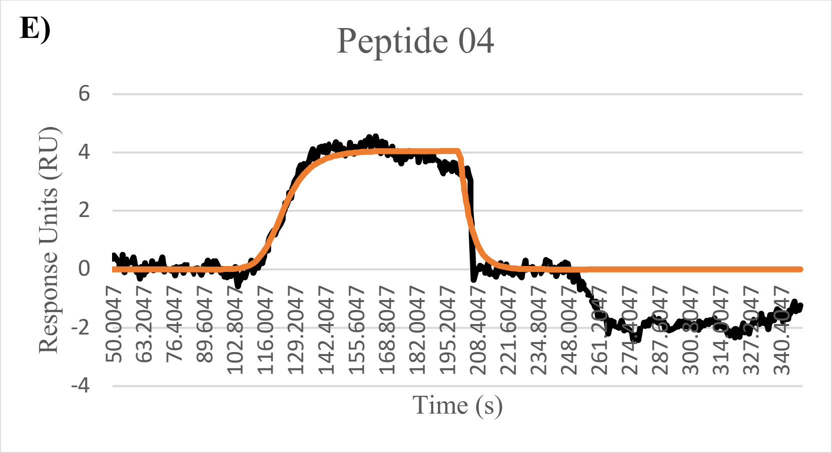
E)
Peptide 04
Response Units (RU)
Time (s)

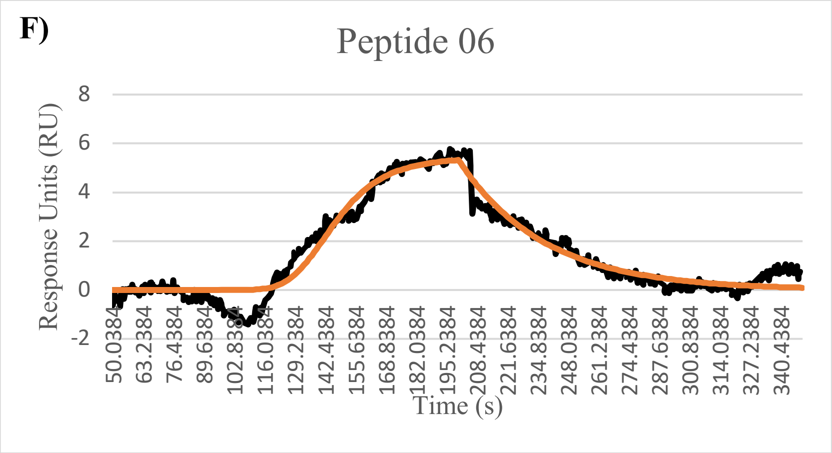
F)
Peptide 06
Response Units (RU)
Time (s)

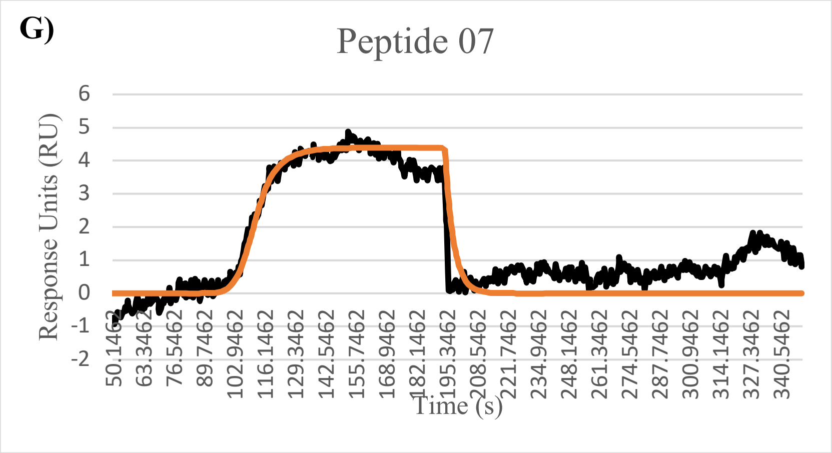
G)
Peptide 07
Response Units (RU)
Time (s)

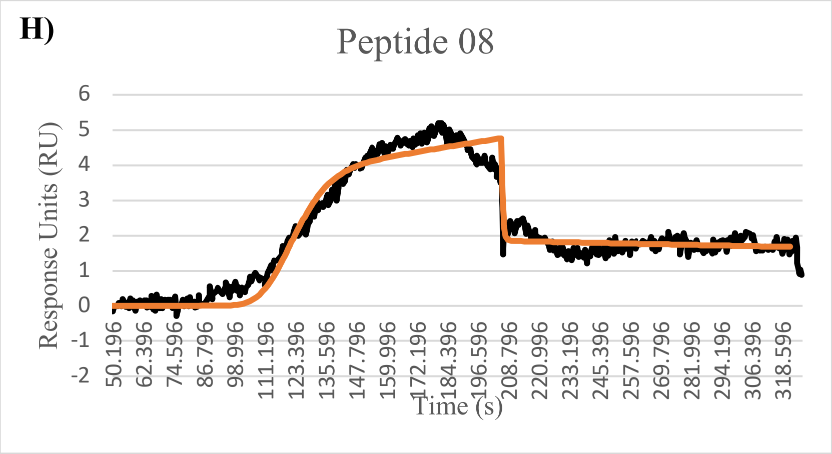
H)
Peptide 08
Response Units (RU)
Time (s)

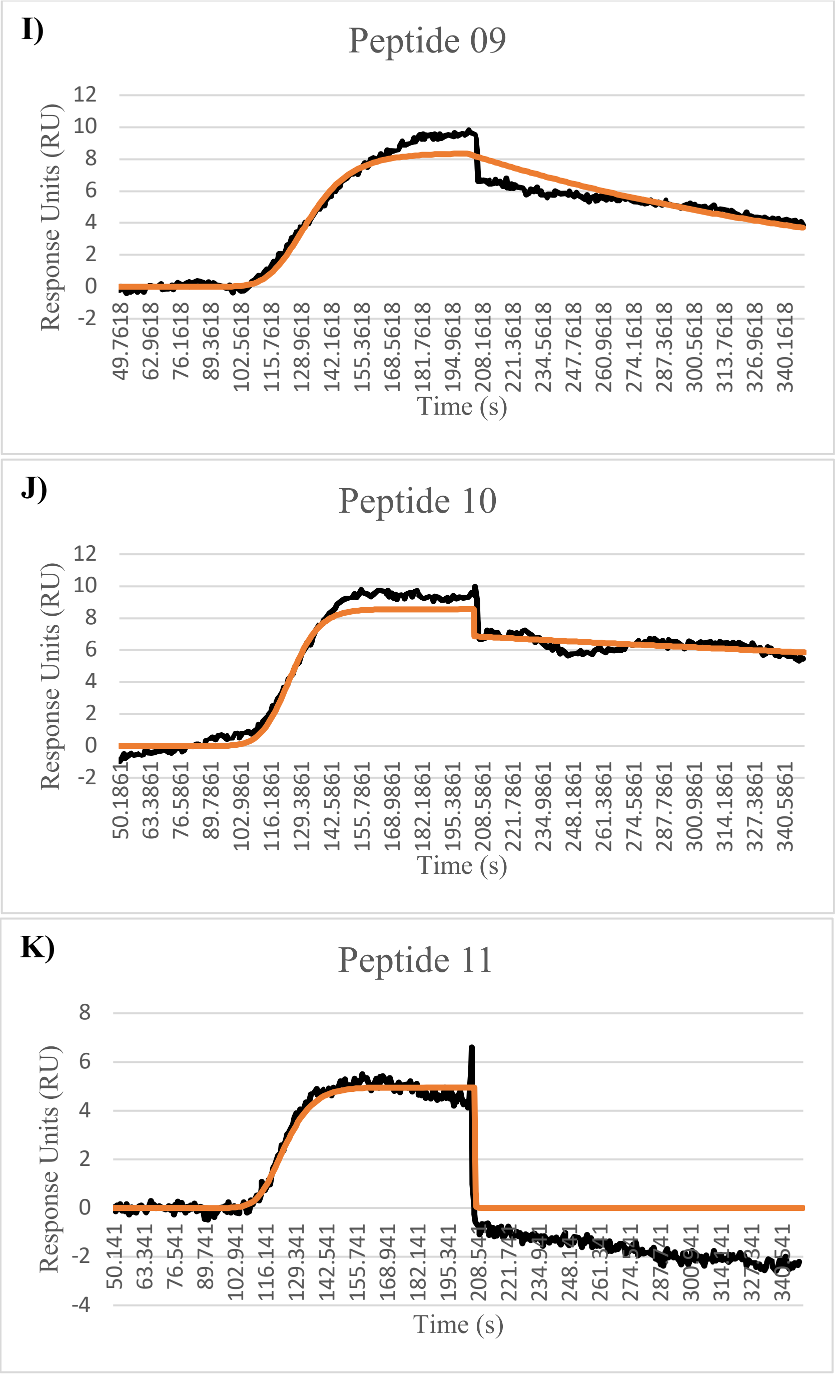
I)
Peptide 09
Response Units (RU)
12
10
8
6
4
2
0
-2
49.7618
62.9618
76.1618
89.3618
102.5618
115.7618
128.9618
142.1618
155.3618
168.5618
181.7618
194.9618
208.1618
221.3618
234.5618
247.7618
260.9618
274.1618
287.3618
300.5618
313.7618
326.9618
340.1618
Time (s)
J)
Peptide 10
Response Units (RU)
12
10
8
6
4
2
0
-2
50.1861
63.3861
76.5861
89.7861
102.9861
116.1861
129.3861
142.5861
155.7861
168.9861
182.1861
195.3861
208.5861
221.7861
234.9861
248.1861
261.3861
274.5861
287.7861
300.9861
314.1861
327.3861
340.5861
Time (s)
K)
Peptide 11
Response Units (RU)
8
6
4
2
0
-2
-4
50.141
63.341
76.541
89.741
102.941
116.141
129.341
142.541
155.741
168.941
182.141
195.341
Time (s)

L)

Peptide	Sequence	K_D (μM) Affinity
MBP-wt	SASALAKIEEGK	8.01 ± 1.40
01	GGGTWAAFEEGK	10.31 ± 2.90
02	VGGGTWAAFETL	12.81 ± 2.55
03	GGTWAAFNDV	8.71 ± 2.14
04	VGGGTWAAIEEGKL	8.83 ± 2.59
05	GGTWAAFE	ND
06	GGGTWAAIE	17.33 ± 0.20
07	TWAAFN	5.38 ± 0.20
08	TWAAIE	0.70 ± 0.35
09	VGGGTWAAIE	1.35 ± 0.41
10	GGTWAAIE	0.89 ± 0.07
11	AAFE	18.5 ± 0.62

Supp. Figure 1: **SPR Sensorgrams from the Kinetic Analysis of Various Peptides with LepB.** Sensorgrams are a representative of triplicate data. The black lines are the Onestep response for the various peptides and the orange lines are the fit curve used by the Qdat software to calculate on- and off-rates (see Table 1). **A)** The MBP-wt (SASALAKIEEGK) peptide with immobilized LepB. **B)** Peptide 01 (GGGTWAAFEEGK) with immobilized LepB. **C)** Peptide 02 (VGGGTWAAFETL) with immobilized LepB. **D)** Peptide 03 (GGTWAAFNDV) with immobilized LepB. **E)** Peptide 04 (VGGGTWAAIEEGKL) with immobilized LepB. **F)** Peptide 06 (GGGTWAAIE) with immobilized LepB. **G)** Peptide 07 (TWAAFN) with immobilized LepB. **H)** Peptide 08 (TWAAIE) with immobilized LepB. **I)** Peptide 09 (VGGGTWAAIE) with immobilized LepB. **J)** Peptide 10 (GGTWAAIE) with immobilized LepB. **K)** Peptide 11 (AAFE) with immobilized LepB. **L)** Affinity of various peptides to LepB calculated using SPR. The mean KD ± SE is from triplicate data.

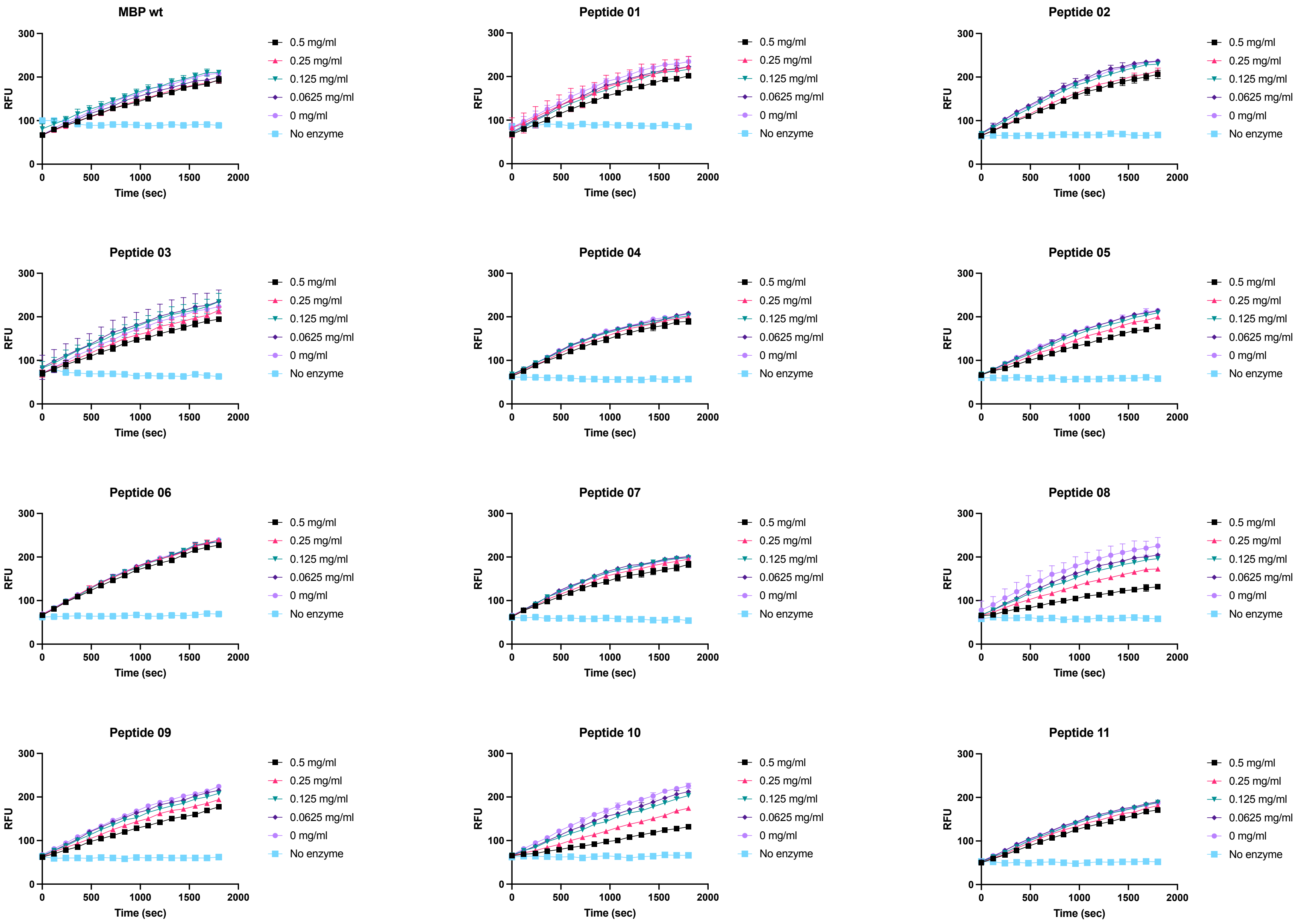

<u>Supp. Figure 2:</u> LepB kinetic enzyme assay in the presence of indicated concentrations of peptides.

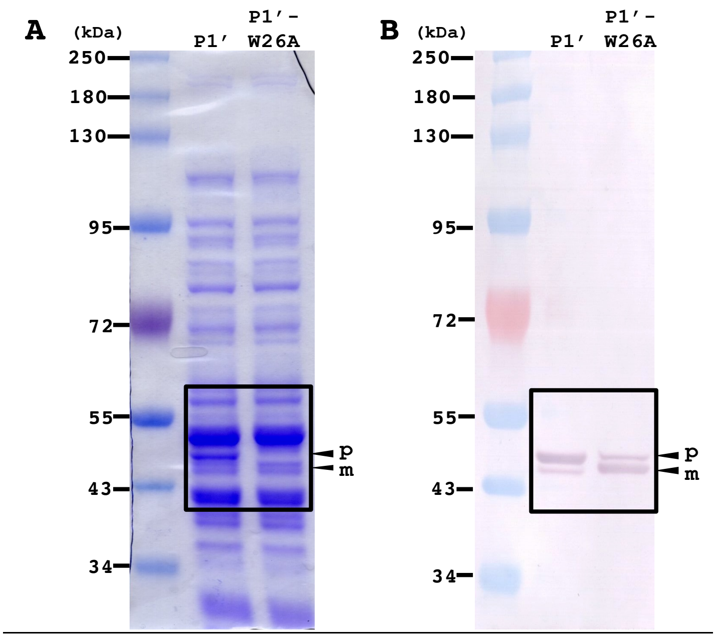

Supp. Figure 3: Full size A) Coomassie stain and B) anti-MBP Western blot of whole cell lysate of TasA-MBP-P1′ and TasA-MBP-P1′-W26A after 30 minutes induction with IPTG. black box = cropped area for Figure 4B Western blot

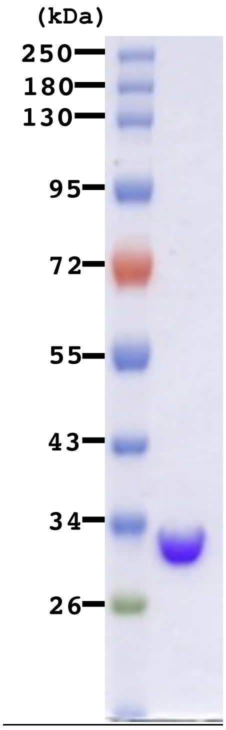

Supp. Figure 4: Coomassie stain of purified untagged LepB Δ2-76
